# Consensus Development Project (CDP): An overview of staffing for safe and effective nursing care

**DOI:** 10.1002/nop2.989

**Published:** 2021-07-18

**Authors:** Jane E. Ball, Peter Griffiths

**Affiliations:** ^1^ School of Health Sciences University of Southampton Southampton UK; ^2^ National Institute for Health Research Applied Research Collaboration (Wessex) Southampton UK

**Keywords:** hospital, nurse staffing, patient safety, policy, workforce

## Abstract

We present an overview of the research evidence on nurse staffing levels in acute hospitals, and how it has been applied to policy and practice, focussing primarily on the UK. Drawing on research reviews and examples of specific studies, we outline the current state of knowledge. Much of the evidence comes from cross‐sectional studies. More recently, longitudinal studies allow a causal link between staffing and outcomes to be inferred. Lack of specificity on staffing levels has hindered application of research findings to practice; research rarely specifies how many nurses are needed for safe and effective care. The most significant impediment to achieving safe staffing has been an underestimation of the number of RNs needed and overestimation of the potential for substitution, resulting in low baseline staffing and a national shortage of RNs. Repeatedly, new staffing solutions are sought rather than tackle the problem of too few RNs head‐on.

## 1 INTRODUCTION AND AIM

This paper was prompted by an invitation by the Royal College of Nursing Strategic Research Alliance to produce an overview of the research evidence on nurse staffing levels, the strengths and weaknesses, and how evidence has been applied to policy and practice. It was undertaken to contribute to a Consensus Development Project, which sought to give members of the public access to expert overviews of research related to nursing. The goal was to outline the essential elements required for safe and effective nursing care provision for adults, to enable discussion and Consensus on next steps.

## 2 BACKGROUND

The basic principle that healthcare providers must have sufficient nursing staff on duty to provide care safely and effectively is enshrined in various guidance and regulations influencing the National Health Service (NHS) in the UK. The NHS Constitution (first published in 2009, and updated several times since) makes explicit that patients have the “*right to be treated with a professional standard of care, by appropriately qualified and experienced staff, in a properly approved or registered organisation that meets required levels of safety and quality*” (DHSC, 2019). NHS organizations are expected to ensure that staffing is sufficient to fulfil this patient right, and staff are expected to raise concerns if conditions for safe care are not in place. This expectation is underpinned by a legal duty for NHS organizations to “*encourage and support all staff in raising concerns at the earliest reasonable opportunity about safety, malpractice or wrongdoing at work, responding to and, where necessary, investigating the concerns raised and acting consistently with the Employment Rights Act 1996*.”

Similarly, the Nursing & Midwifery Council (the body that regulates nurses and midwives in the UK) is explicit about registrants’ professional obligations to raise concerns if staffing levels are insufficient. The Code instructs: “*You must act without delay if you believe that there is a risk to patient safety or public protection*.” (NMC, 2018).

However, the specifics of what constitutes “safe staffing” levels are not explicitly stated in the UK. Each of the four nations in the UK has different situations, in terms of guidance and legislation to ensure that staffing levels are sufficient for safe and effective care. (RCN, 2017).

In England, decisions about the number and mix of nurse staffing required to be employed or deployed in a particular clinical area are taken at a local level by individual NHS Trusts. There is no national mandate that sets out what that level should be. The regulator (Care Quality Commission) is duty‐bound to refuse to register a healthcare provider if they cannot satisfy them that they comply with Regulation 18 to “*provide sufficient numbers of suitably qualified, competent, skilled and experienced staff to meet the needs of the people using the service at all times*.”(CQC, 2014) But it is up to the care provider to judge what constitutes “sufficient,” and decide how to assess the nurse staffing needed for a particular service.

The care crisis identified by unexpectedly high mortality rates in an English acute hospital (Mid Staffordshire Trust) exposed the risks associated with not having clear guidance on nurse staffing levels. Sir Robert Francis (QC) undertook a forensic examination of the factors contributing to what became evident was an appalling degradation of care services at the Trust. (Francis, 2010) Nurse staffing levels that were insufficient—and that had been reduced in order to cut costs—were identified as one factor. A second inquiry (published in 2013) looked at how it was possible that a hospital in a *national health service*—with national systems and regulation—could have fallen so far below expected standards, without problems being detected. (Francis, 2013) Wider system failings were identified, and recommendations were made, some of which related to nurse staffing.

The inquiry, and subsequent public and media outcry, led to a range of policies being introduced that aimed to remedy the situation. The Government's response included asking the National Institute for Health Care Excellence (NICE) to produce evidence‐based guidelines on safe nurse staffing level for each specialty/setting, starting with acute adult wards in general hospitals.

In 2014, a research team led by Griffiths undertook the review of evidence to support the development of guidelines for acute adult inpatient settings, and reported to the NICE advisory panel. (Griffiths et al., 2014) Safe staffing guidance for nursing in adult general acute hospital wards was issued by NICE later that year.(NICE, 2014) NICE recommended using “red flags” to monitor instances where nurse staffing levels were insufficient to meet patients’ needs. This included instances of marked shortfall, defined as “A shortfall of more than 8 hr or 25% (whichever is reached first) of registered nurse time available compared with the actual requirement for the shift.”(p24); staffing of 8 patients per RN (a level associated with increased risk of harm in the literature) should trigger an urgent review. The research underpinning the guidance on staffing levels and skill mix is described below.

Another element of the government response was the publication in November 2013 of the NQB report, which set out principles that Trusts were expected to apply to ensure they had “the right staff, in the right place, at the right time” (NQB, 2013). The Trust had responsibilities for reviewing staffing and ensuring it was adequate.

Elsewhere in the world (e.g. California, parts of Australia and South Korea) guidance has gone further—to stipulate legally enforceable staffing minimums, calibrated by type of hospital, specialty and time of day/night. Mandatory staffing policies in the USA and Australia suggest minimum staffing levels that are equivalent to between four–seven patients per nurse in general acute wards during daytime (RCN, 2012).

## 3 METHODS AND DESIGN

This is a discussion paper. We have applied our expertise and knowledge to provide an expert overview on the current state of evidence related to nurse staffing, and factors that influence its translation into policy and practice. As we have generated a substantial volume of research in this field over the last 30 years and worked with policymakers, unions and employers to advise on decision‐making in relation to nurse staffing, we cannot ignore our own perspective. Although we aim to highlight the most significant research in the field, our overview is selective and influenced by subjective assessments but seeks to prompt discussion and help determine next steps.

We have focussed on evidence related to staffing in acute hospital wards. There is a lack of research evidence in mental health or community settings. This created a significant challenge for the national panels set up by NICE and then taken over by NHS Improvement (at the instigation of NHS England), aiming to produce guidelines for “safe and effective staffing levels.” However, a number of key papers outside of the physical general acute sector are worthy of note in relation to mental health (Bowers & Crowder, 2012; Cook et al., 2019), nursing homes (Spilsbury et al., 2011) and primary care (Griffiths et al., 2010; Murrells et al., 2015).

No research permissions or Research Ethics Committee approvals were required or sought for this paper.

## 4 DISCUSSION

### 4.1 Research evidence: old and new

A systematic review of research confirming the relationship between low nurse staffing levels and adverse patient outcomes found 101 studies published up to 2006, mainly from the USA (Kane et al., 2007). The review that included a meta‐analysis that pooled data from 28 studies concluded that higher RN staffing levels were associated with lower odds of hospital‐related mortality and adverse patient events.

Since then, the research from other countries has increased, including Australia (Twigg et al., 2011), China (You et al., 2013), England (Rafferty et al., 2007) and across nine European countries (Aiken et al., 2014). In addition to looking at systematic reviews such as Kane's, our review for NICE in 2014 found 35 primary studies that met our strict criteria. (Griffiths et al., 2014) Almost all the studies we identified used cross‐sectional data (survey, or routinely collected data), and most measured associations between staffing and outcomes at a hospital level. Sample sizes varied from studies covering hundreds of hospitals with millions of patients, to single‐centre studies and studies with less than 1,000 patients. Summarizing our findings across these 35 studies, we concluded that there was:

- Strong evidence from several large observational studies that lower nurse staffing levels were associated with higher rates of death and falls.
- Strong evidence that higher nurse staffing is associated with reduced length of stay and lower readmission rates.
- Similar but less consistent evidence on infections.
- Contradictory evidence on pressure ulcers.
- No evidence of an association with venous thromboembolism.

Most of the studies in this field have used a cross‐sectional design; associations between variables are established but we cannot prove causality. Although the causal relationship between staffing and patient outcomes seems probable, there are a number of gaps in some studies that allow for possible bias, and weaken the evidence base overall. Limitations identified in the 2014 review included the following:

- Omitted variables: for example, few studies take account of staffing levels of doctors; some studies do not consider differences in the mix of patients.
- Simultaneity: factors such as acuity that influence outcomes also influence staffing levels at the same time
- Common‐method variance: a reported association could be a result of both things being measured in the same questionnaire.

These limitations in methods (and some inconsistencies in the results in relation to specific outcomes such as pressure ulcers) have led some to question the validity of this evidence. Nonetheless, reviews of the evidence as a whole are broadly consistent with a cause‐and‐effect relationship—that is that the reason we see an increase in negative outcomes when staffing levels are lower is at least in part due to low staffing levels that cause worse outcomes. For example, our findings from the multi‐country RN4Cast Study were reported in the Lancet: an increase in a nurses’ workload by one patient increased the likelihood of an inpatient dying in 30 days of admission by 7%. It is not only the number of nurses that makes a difference, but the level of education. This same study reported that hospitals with higher proportions of degree‐educated RNs had lower levels of patient mortality after common surgery (after taking patient differences into account). The presence of a larger number of less highly trained staff does little to compensate for low RN staffing, because their roles are of course different (Aiken et al., 2016).

Our research since this time has helped to establish the plausibility of a causal link between RN staffing and patient outcomes. We have found that when there are fewer RN staff on duty, necessary care is less likely to be completed in full (Ausserhofer et al., 2014; Ball et al., 2014). Further analysis of data from the RN4Cast study suggests that this “missed RN care” may be part of an explanatory link between RN staffing and patient mortality in hospital (Ball et al., 2017). After common surgical procedures, patients are more likely to die as a result of the care they receive (not their condition or comorbidities) if they are in a hospital with higher level of missed care: each additional 10% of missed care (calculated using a 13‐item scale that nurses completed) is associated with a 16% increased risk of patient death.

Increasingly, this conclusion—that staffing is causally linked to outcomes—is confirmed by longitudinal studies that link individual patients to daily or even shift level staffing (Griffiths et al., 2019; Needleman et al., 2011; Shang et al., 2019). The first of these was a seminal study by Needleman and colleagues published in 2011 (Needleman et al., 2011). Nurse staffing was measured through routinely collected administrative data and was thus recorded for every shift, covering 176,696 eight‐hour shifts from 43 units in one hospital. A statistically significantly increased risk of mortality was observed *after* periods of exposure to low staffing. The study made explicit an objective measure of “low staffing” rather than relying on a relative measure of RN staffing. Risk of patient death increased after exposure to RN staffing deficit—that is shifts where the number of nursing hours per patient day (NHPPD) is at least eight hours fewer than estimated as required (i.e. a shortfall of one RN).

In 2019, we published findings from a retrospective longitudinal observational study in the NHS, which used routinely collected data over a three‐year period to capture daily staffing levels and patient mortality. (Griffiths, Ball, et al., 2018; Griffiths et al., 2019). The hazard of death was increased by 3% for every day that a patient experienced RN staffing below the ward average. Importantly, although low nursing assistant staffing was associated with increases in mortality, high nursing assistant staffing was *also* associated with increased mortality. The findings highlight the possible consequences of reduced nurse staffing and highlight potential risk of policies that encourage the use of nursing support staff to compensate for shortages of RNs.

These studies avoid many of the limitations of cross‐sectional studies and provide even stronger evidence that some avoidable adverse outcomes for patients are caused by deficits in care that occur when nurse staffing is low. The newer evidence supports the plausibility of a causal mechanism linking nurse staffing levels to patient outcomes: low nurse staffing limits the ability of nurses to deliver high‐quality care, which can lead to low job satisfaction, errors or omissions in care and, in some cases, adverse outcomes for patients (Griffiths, Recio‐Saucedo, et al., 2018; Recio‐Saucedo et al., 2018).

This growing and strengthening evidence base elucidates the risks associated with low RN staffing levels and highlights the benefits of higher nurse staffing.

However, despite the strength of the evidence to support the general association, this evidence offers little direct guidance to those wishing to set staffing levels on wards. Most studies simply offer an estimate of the average effect of changing staff levels, without reference to the actual levels. The estimates give no clear indication of the actual staffing levels to be achieved. In effect, the answer to the question of what staffing level is needed is that “higher” is better than “lower.” Our review for NICE also noted the limited evidence for tools used to determine staffing requirements, a finding that was confirmed in more recent reviews. (Griffiths, Saville, Ball, Jones, et al., 2020; Saville et al., 2019) This is one of the challenges that prevented NICE from being able to stipulate with more certainty what a “safe minimum” level of RN staffing would be for general acute wards.

### 4.2 NHS/UK‐based evidence to inform policy and practice in the UK

While the relationship between nurse staffing and outcomes has been observed in a diverse range of countries, differences in the configuration of services and composition of the workforce make it unlikely that the same staffing level would apply in all settings. We therefore collated NHS general ward‐based evidence for NHS Improvement, to create a resource to support “safe and sustainable” staffing decisions in the NHS. (Griffiths et al., 2017) In this review, we selected studies undertaken in the UK that estimated associations between nurse staffing levels on general wards and any quality or outcome measure.

Ten papers that reported associations between nurse staffing levels and outcomes in the NHS, published between 1999–2016 using data gathered from 1992–2010, were identified. These papers related to seven distinct research studies, all of which adopted an observational design. Samples were typically large, ranging from 2917–8887 nurses and from 9877–over 12 million patients. Similar to the broader NICE evidence review, most analyses in most studies showed a statistically significant difference between nurse staffing and outcomes including mortality, staff burnout and incomplete nursing care. (Shuldham et al., 2009).

Five papers derived from three distinct studies reported associations between specific ward‐based staffing levels and some measure of quality or a patient or nurse outcome in the NHS. The odds of death for surgical patients were increased by 26% in the hospitals with lowest staffing on general wards (over 12 patients per RN, hospital‐wide) compared with the best (8.4 patients per RN or fewer) (Rafferty et al., 2007). For medical patients, the odds of death were reduced by 11% in hospitals where the average staffing on medical wards was 6 or fewer patients per RN (Griffiths et al., 2016). A similar association was seen for surgical patients in surgical wards, but it was not statistically significant. Stroke units with 6.7 or more beds per RN on weekdays had 31% higher mortality compared to units with 3.3 or fewer beds per RN on weekdays (Bray et al., 2014). The difference was even greater for weekend staffing levels.

Nurses’ reports of poor or declining quality were more likely in hospitals with the lowest staffing on general wards (12+ patients to nurse) compared with the highest (8 patients or fewer per nurse) (Rafferty et al., 2007). The odds of nurses reporting missing necessary care were reduced by 66% in better staffed wards (≤6 patients per RN) compared with the worst (11+ patients per RN) (J. E. Ball et al., 2014).

Odds of reporting dissatisfaction and emotional exhaustion were reduced by 43% and 30% among nurses in the best staffed wards (≤4 patient per RN) compared with the worst (13+) staffed wards (Sheward et al., 2005).

Five of these studies considered the relationship between support worker or healthcare assistant staffing and outcomes in their analysis in addition to RN staffing. In three of these, there was some indication that higher levels of support worker staffing or lower skill mix were associated with worse outcomes, although studies reporting relationships with HCAs deployed on wards found no association (positive or negative) with the outcomes studied.

While the odds of adverse outcomes were generally increased when average staffing fell below the 1:8 level, better outcomes were often associated with higher staffing levels and ratios of 1:7 and lower. For some services, significant increases in risk occurred well below this threshold. While not giving a clear “safe” staffing level, this evidence reinforced the guidance issued by NICE—that a 1:8 represents a level at which risk is known to be increased and would not represent an optimal safe staffing level.

While the evidence from the NHS shows that lower nurse staffing levels are associated with worse outcomes, it is once again hard to discern a clear threshold of sufficiency. In many studies, the staffing levels reported are averages, and in several studies, the statistically significant differences in outcomes were reported when comparing the best to the worst staffed wards.

Our most recent research—an in‐depth study in a single NHS hospital Trust—takes the effort to quantify the staffing and outcome relationship one step further (Griffiths, Ball, et al., 2018). In this study, we explicitly tested for and found a linear relationship between patient‐level exposure to staffing at different levels and benefits. However, no “threshold” effect was found; patient benefits increased in proportion to increased hours of nursing input, without reaching a discernible “plateauing” effect. The finding is important as it suggests that the current range of staffing levels we observe in the UK do not actually reach an “optimum” level; we cannot assume that current “norms” (even when all posts are filled) are optimal.

### 4.3 Applying evidence to practice: considerations

Providing robust guidance on “safe and effective” staffing is not without its challenges. It is not possible to determine “safe and effective” staffing levels without taking the following three factors into account:

1. Care for whom? Patient needs vary: differences in terms of patient needs for nursing care (dependency), levels of sickness (acuity) that make a difference to the staffing level required, and skill mix (i.e. balance between RN and support staff, and proportion of higher grade staff with more experience, and clinical expertise).
2. Who's providing nursing care? Differences in how care is provided and the composition of the team providing nursing care: not only between places in the UK where such guidance would be applied but also between countries where research evidence has come from. Most of the evidence we have described is specific to Registered Nurse staffing levels. Where other staff groups were considered, there was no evidence to support substitution of healthcare assistants for RNs. The required levels of HCA staffing are unclear from this evidence and must be determined in addition to RN staffing levels.
3. In what context? Studies have shown that the environment—in terms of factors such as nursing leadership, relationship between staff, communication—also contributes to the effectiveness of staffing. The “right” nurse staffing in a poor practice environment is unlikely to achieve good‐quality care.

Minimum ratios can provide a “safety net” or warning level to trigger review, but ultimately, the exact number and profile of staff needed to deliver care safely, and effectively, has to be determined locally, using workload assessment tools (such as the “Safer Nursing Care tool” endorsed by NICE), and adhering general principles of triangulation (examining staffing need from multiple angles), with regular review and monitoring. Recent research from our group has added to the limited evidence about the validity and effectiveness of workload assessment tools that was identified by NICE, but strongly reinforces the importance of using professional judgement alongside measurement. (Griffiths, Saville, Ball, Chable, et al., 2020; Saville et al., 2020).

The policies set out following Francis were designed to support improving safe staffing in the NHS.

We were commissioned to evaluate how the national safe staffing policies introduced after Francis had been implemented in NHS acute hospitals and whether evidence‐based guidelines and other policies made a difference to safe staffing in practice. (Ball et al., 2019) Based on an analysis of national workforce data, a national survey of Directors of Nursing in all acute hospital Trusts, and case studies, we found a number of positive changes: new approaches to staff planning and rostering, and increased board awareness of safe staffing. The number of nursing staff employed in the NHS acute sector increased since 2013 by 10% for Registered Nurses (RNs) and 30% for support staff (HCAs). However, numbers also increased so the goal of safe staffing had not been consistently achieved; 25% of Trusts reported the RN per number of patients routinely exceeded 1:8. The biggest challenge to achieving safe staffing was difficulty filling posts; the average RN vacancy rate reported was 10%. External pressures (lack of workforce supply and reduced access to temporary staffing) had constrained Trusts’ abilities to fully implement policies aimed at ensuring safe staffing on acute wards.

So while the safe staffing policies introduced after the Francis Inquiry had provided leverage—raising the profile of nurse staffing at board level—a lack of “joined‐up” policy at a national level and failure to grow the Registered Nurse workforce had prevented Trusts from fully achieving the government vision following the Mid Staffordshire of putting “Patients First and Foremost” (Health, 2013) by having safe staffing on every NHS ward.

### 4.4 Implications

Finally, we end this overview with a summary of safe and effective staffing in context. Key points to note in interpreting the evidence, the policies and the implementation of policy are as follows:

- On many wards in the UK, much of the direct nursing care is provided by healthcare assistant staff who have had an average of two‐week training to prepare them for their roles (Arthur et al., 2017).
- The research evidence demonstrates that the number of RNs (and their qualification levels) makes a difference.
- The vast majority of guidance and policies produced centrally in the UK have been non‐specific, both in terms of who provides care (and the mix between RNs and support staff) and number of RNs required. Repeatedly, principles of good HR management and workforce deployment are espoused but without specific levels being referenced. The RCN has nonetheless issued its own guidance that is more specific, for example referencing staffing ranges for elderly care settings in hospitals (Hayes & Ball, 2012), and guidance that RNs should constitute at least 65% of the nursing team in acute settings (RCN, 2006).
- Even in the parts of the UK where safe staffing legislation has been introduced (Wales and Scotland), it focusses on the following best practice or mandating application of workload assessment. In Northern Ireland, “normative ranges” have been identified, but are not mandated.
- We lack specialty‐specific standards against which to measure achieved staffing level, or to ensure a safe minimum is always present.
- There is a national shortage of Registered Nurses. For the past decade, RNs have been in short supply and on the occupation shortage list. Globally, WHO estimates we will need an additional 9 million nurses and midwives by the year 2030 (WHO, 2020).
- Parts of the UK have relied on recruiting from overseas to plug the gaps, but this is a short‐term option that fails to address the longer term problems of not educating enough RNs to meet healthcare demand in the UK (in NHS and other sectors such as care homes, private).

The widespread national shortages reveal that for many years, there has been a consistent failure to train enough nurses in the UK (Buchan et al., 2020). The growing demand for health care, low baseline staffing levels and unwarranted assumptions about the ability of support workers to substitute for Registered Nurses have led to a repeated underestimation of the training places needed, and resulted in an ongoing national shortage.

## 5 CONCLUSION

The evidence described in this paper demonstrates the potential consequences of shortages of Registered Nurses on duty in terms of adverse patient outcomes including, but not limited to, the risk of death. The research evidence suggests that the shortage of Registered Nurses faced in the UK is unlikely to be ameliorated by increased provision of support workers, even though improving the training of such staff is, in itself, to be welcomed. Substitution of less qualified staff for Registered Nurses is unlikely to be either safe or cost‐effective. Solutions to the shortage of Registered Nurses requires the undersupply of RNs, and factors that have led to it, to be addressed.

The long‐term solution to achieving safe staffing is not just about training enough nurses to allow safe staffing level to be achieved, but about seeing the value in *having* enough, to ensure we have sufficient supply and that we provide working conditions that can retain and develop the nursing workforce, to meet UK’s current and future health needs.

## CONFLICT OF INTEREST

Neither author has any conflict of interest to declare.

## AUTHOR CONTRIBUTIONS

Both authors have fully met criteria of authorship, having: made substantial contributions to conception and design of the paper; been involved in drafting the manuscript and revising it been given final approval of the version submitted to be published; participated sufficiently in the work to take public responsibility for the content; and agreed to be accountable for all aspects of the work in ensuring that questions related to the accuracy or integrity of any part of the work are appropriately investigated and resolved.

## ETHICAL APPROVAL

Ethical consent was not needed and not sought (as this is a discussion paper).

## INFORMED CONSENT

Patients were not involved so no patient consent was required.

## Data Availability

The authors are requested to include a data accessibility statement, including a link to the repository they have used, in order that this statement can be published alongside their paper. In this case, as the paper is a discussion of the issues arising from published research—which is fully referenced—plus expert opinion form the authors themselves, there are no additional data to make available, but the lead author would be happy to receive any questions or correspondence related to the paper from readers.
